# Unveiling the last missing link of the cardiolipin synthetic pathway in mitochondria

**DOI:** 10.18632/aging.100572

**Published:** 2013-06-26

**Authors:** Yasushi Tamura, Toshiya Endo

**Affiliations:** ^1^ Research Center for Material Science; ^2^ Department of Chemistry, Graduate School of Science; ^3^ Structural Biology Research Center; ^4^ Core Research for Evolutional Science and Technology (CREST), Nagoya University, Chikusa-ku, Nagoya 464-8602, Japan

Mitochondria produce cellular energy ATP by using electrochemical energy stored as a proton gradient across the mitochondrial inner membrane (IM). To generate the proton gradient, the respiratory-chain complexes in the IM pump up protons from the matrix to the inter-membrane space by coupling it to the electron transport through the respiratory chain. The efficient electron flow relies on the respiratory-chain super-complex formation, which is facilitated by the mitochondrial signature phospholipid cardiolipin (CL) [1]. CL directly interacts with the respiratory-chain complexes, mitochondrial carrier proteins including ADP/ATP carrier (AAC) etc. and stabilizes the super complexes consisting of Complexes I, III and IV in mammals and Complexes III and IV with AAC in yeast [1]. In addition, it is known that the assembly and maintenance of the TIM23 complex, a mitochondrial protein translocator in the IM, depends on the presence of CL [2].

Although the electron transport is essential for energy production in mitochondria, it could also generate reactive oxygen species (ROS), which are the potential cause of the oxidative damage of DNA and proteins as well as phospholipids in mitochondria. Accumulation of oxidative damage is thought to lead to mitochondrial and cellular dysfunction associated with aging [3]. CL is one of the main targets of ROS in mitochondria due to its IM location close to the sites of ROS production and its feature containing high-levels of unsaturated fatty acids [3]. Therefore CL could be a key molecule in aging process.

CL is synthesized through several steps of modifications of phosphatidic acid (PA) in the IM and is remodeled by deacylation and re-acylation that generate mature CL with unsaturated fatty acids [1]. Despite its physiological importance, it was not clear how CL is synthesized in mitochondria. In particular, an enzyme responsible for the first step of the CL synthesis, i.e. generation of an important intermediate phospholipid, CDP-diacylglycerol (CDP-DAG), in mitochondria was not identified.

**Figure 1:**
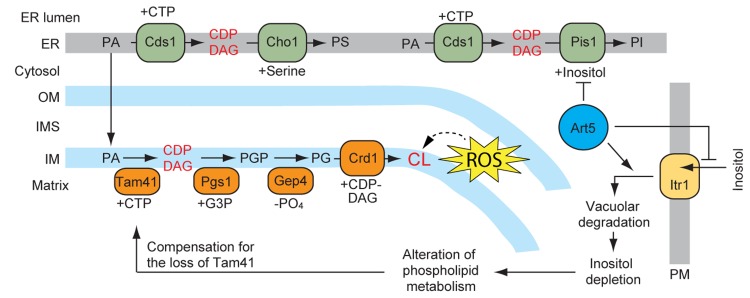
Roles of Tam41 and Art5 in yeast hospholipid biosynthetic pathways PM, plasma membrane; ER, endoplasmic reticulum; OM, mitochondrial outer embrane; IMS, mitochondrial intermembrane space; PS, phosphatidyl serine; PGP, phosphatidyl glycerol phosphate; PG, phosphatidyl glycerol.

In the issue of Cell Metabolism (Volume 17, Issue 5, 709-718, 7 May 2013), we reported identification of Tam41 as a mitochondrial CDP-DAG synthase and the cellular system to compensate the loss of Tam41. Tam41 was originally identified as a protein important for assembly and maintenance of the TIM23 complex in the IM [4]. However, a subsequent study showed that loss of Tam41 leads to significant decrease in CL and accumulation of PA, the precursor phospholipid of CDP-DAG [5]. Besides, respiratory-chain super complexes tend to be destabilized in cells lacking Tam41, which indicates that the role of Tam41 is not limited to the TIM23 complex but to the protein complexes in the IM in general [5]. We thus reasoned that Tam41 could be a mitochondrial CDP-DAG synthase and asked whether Tam41 directly catalyzes the CDP-DAG formation. To this end, we purified Tam41 and its loss-of-function mutants from yeast cells and measured their CDP-DAG synthase activities in vitro. We observed generation of CDP-DAG upon incubation of wild-type Tam41, but not the Tam41 mutants, with fluorescence-labeled PA and CTP. These results demonstrate that Tam41 is a mitochondrial CDP-DAG synthase, which had been elusive previously (Figure 1).

Our previous study identified Art5 as a multi-copy suppressor of *tam41*Δ cells [6]. Art5 is a member of the arrestin-related trafficking adaptor (ART) protein family and is known to facilitate degradation of inositol transporter in plasma membrane in response to exogenous inositol [7]. Indeed, we found that growth defects of tam41Δ cells were restored in inositol-depleted media (Figure 1). Surprisingly, despite the restoration of the growth defects of *tam41*Δ cells by overexpressing Art5, the low level of CL hardly changed while the CDP-DAG, PA, and PE (phophatidylethanolamine) levels increased and the PI (phosphatidylinositol) level decreased. Hence CL itself is not critical for cell growth, but overall alteration of the phospholipid composition is capable of compensation of the loss of Tam41. This observation also points to the fact that yeast cells have a backup system to tolerate the loss of Tam41. In relation to this, Connerth et al. showed that defects in cell growths and decrease in the CL level caused by the loss of Tam41 can be largely restored by the simultaneous loss of a PA transfer protein Ups1 [8]. This suggests the presence of an alternative CL synthetic pathway, which may be activated in the absence of both Tam41 and Ups1. These findings may offer hints about possible treatments of diseases and protection against aging processes associated with mitochondrial dysfunction arising from the compromised CL synthesis or CL oxidation.
